# The Correlation between Social Support and Quality of Life of Seniors without Cognitive Disorders from an Institutional Environment—A Descriptive Cross-Sectional Survey

**DOI:** 10.3390/healthcare8030212

**Published:** 2020-07-14

**Authors:** Paweł Chruściel, Beata Dobrowolska

**Affiliations:** Department of Basic Nursing and Medical Teaching, Faculty of Health Sciences, Medical University of Lublin, 4 Staszica Street, 20-081 Lublin, Poland; bb.dobrowolska@gmail.com

**Keywords:** quality of life, social support, older adult, institutional care, nursing home, residential care home

## Abstract

The aim of this study was to confirm the correlation between social support and the quality of life of seniors who live without cognitive disorders, but are living in an institutional environment. The measurement of variables was based on a cross-sectional survey method. A representative sample of 957 seniors participated in the survey. The study covered public nursing homes (NHs) and residential care homes (RCHs) selected for the study by stratified sampling. The results confirmed a linear correlation between social support and the quality of life of seniors (*p* < 0.001). It was also confirmed that there were differences in the range of studied variables depending on the type of institution (NH versus RCH; *p* < 0.001). Social support is a significant component, and at the same time, a modifier of the perception of the quality of life for older people. Its variable level determines the subjective assessment of functioning in particular domains of quality of life. RCH as an institution of a social nature satisfies the needs for support at a higher level than NH, which translates into a better perception of older people’s quality of life.

## 1. Introduction

Ageing societies, combined with the growing needs and requirements for long-term care and social support, are among the key tasks for health care providers. The older people belong to the most expensive and most difficult recipients of health care services [1]. This results from the fact that they are more likely to suffer many diseases and disabilities. Poland is one of the countries where older people are primarily cared for by members of their immediate families (most often children). Traditions in home care are connected with the need to keep seniors in a natural environment with their families, for as long as possible. However, the functions of a family have been changing with modernisation [2,3]. There is a gradual, but noticeable, decline in the level of commitment towards the realisation of care functions. This tendency is also caused by insufficient care abilities, such as small families, poor families, or long distances between individual members of the family network. For this reason, seniors perceive the institutionalisation of care as an extremely negative phenomenon that often leads to social exclusion and stigma [2].

The role of social support in the context of self-assessment of the older people’s quality of life has been well documented in the literature [2,3,4,5,6,7,8,9,10,11,12,13,14,15,16,17]. It has been confirmed that older people tend to suffer from a lack of social support, which directly contributes to a reduction in their quality of life. The needs of seniors are usually complex, as health deficits of the older people almost always accumulate with social determinants of life, such as social support, education level or sex [18]. This plays a determining role in the deterioration of their life situation, both in terms of health and social relations. Deterioration of self-care abilities and cognitive disorders intensify the demand for services of a nursing and caring nature. Apart from the undeniable correlation between social contacts and relations with health status, they also have a significant impact on the quality of life of seniors [18,19]. In this current study the subjective aspect of quality of life is highlighted, which can be assessed only by the respondent himself. Assessing the level of satisfaction of the respondent is associated with capabilities of his functioning in state of health and illness. Therefore, the quality of life can be understood as the satisfaction with the degree of fulfilment of needs in the context of biological, mental, social and cultural determinants [20].

When assessing the quality of life among people with chronic diseases, support is considered as one of the most important variables [3,11]. Social support is a subjective perception of satisfaction with personal contacts. According to Andrew [5], social support is a kind of interpersonal transaction during which emotions, information, values or instruments can be exchanged. Strong and positive relations with significant others are particularly important when dealing with stressful events. A change in the living environment or being in a new environment reduce our internal resources such as: Sense of being in control, self-esteem or sense of purpose in life. Support allows one to—at least partially—compensate for shortages [6,7,8,9]. It can be assumed that all types and forms of support, especially emotional support, are desirable in unfavourable situations [19,20]. This type of support is particularly important for seniors who, for various reasons, have been deprived of natural sources of support. It has a beneficial effect on mental health and the quality of life, it increases self-esteem and is a source of emotional stability.

The physiological consequences of old age lead to disability and a dependence on other people. The dependence is permanent and increases over time [14,21,22]. With ageing, the number of available sources of support decreases [9,13,14,15]. An increase in demand for long-term care during this period of life requires the involvement of professional carers. Professional care is considered to be a less valuable form of help to the older people, as it does not create a sense of stability and security, unlike family members [12,14]. However, due to the impaired care functions of the family, our societies are increasingly confronted with situations where assistance in the form of institutional support in everyday life becomes necessary.

Residential care homes—provides live-in accommodation, with 24 h-a-day supervised staffing for older/disability people, who may need additional help with their personal care (e.g., dressing, personal hygiene, medication, communication, mobility and feeding). NHs equally provide 24 h care and support, but with added nursing care and assistance for residents who require supervision by a qualified nurse (e.g., devise and monitor care plans, provide and administer treatment, carry out medical interventions). In general, NHs are intended for people requiring special medical care during their stay [4,5,12]. Providing an older people with round-the-clock care of an institutional nature involves a change in their living environment. As a consequence, it leads to a decrease in the quality of life and deterioration of social support, regardless of the type of institution [10,11]. Moreover, isolating an older people from natural support networks may have a negative impact on their health, including their mental performance [19].

For that reason, we focused on older people living in NHs and RCHs. It was difficult for us to find publications describing the quality of life and social support provided for seniors living in NHs and RCHs. The vast majority of scientific contributions were based on a synthesis of data from long-term care or separately from NHs/RCHs. This prevented us from making specific comparisons between the two types of care institutions. However, considering the differences between these two types of care institutions, we have attempted to formulate and verify Hypothesis 2.

This study aimed to confirm the correlation between social support and the quality of life of seniors who live without cognitive disorders, but are living in an institutional environment. The following hypotheses were verified on the basis of the stated aim:

**Hypothesis** **1.** *Quality of life remains dependent on social support. A higher level of social support corresponds to a better quality of life.*

**Hypothesis** **2.** *The type of care institution determines the perception of the quality of life and social support. Social support and quality of life are better assessed by seniors staying in RCHs than in NHs.*

## 2. Materials and Methods

### 2.1. Respondents

A sample of 957 seniors participated in the study. It was determined on the basis of a government officials approved sampling framework. All respondents stayed in long-term care institutions:

- NH—473 respondents;
- RCH—484 respondents.

Three provinces of the Eastern Poland (Lubelskie Province, Podkarpackie Province and Podlaskie Province) was chosen as the study area (general population) because they have one of the highest percentages of older people in the Polish population.

Respondents that participated in the study were selected out of the total number of 142 public care institutions at random—a non-returnable and proportionate—stratified scheme. Seniors from 71 care institutions were studied (38 NHs and 33 RCHs). Prior to selecting the respondents, seniors in psychiatric care, seniors with cognitive disorders and those who did not give consent to participate in the survey were removed from the general population. Then, the maximum acceptable error of the estimate (4%) and the confidence factor of 95% (*p* < 0.05) were determined. The sample size was determined separately for seniors staying in NHs (*n* = 481) and RCHs (*n* = 523) using the on-line formula for the minimum sample size. Data was collected for a period of 6 months by a trained interviewers—caregivers and nurses employed in the studied institutions. The task of the interviewers was to select seniors according to established inclusion criteria, distribute sets of questionnaires and possibly help in understanding the content of the questions.

**Figure 1:**
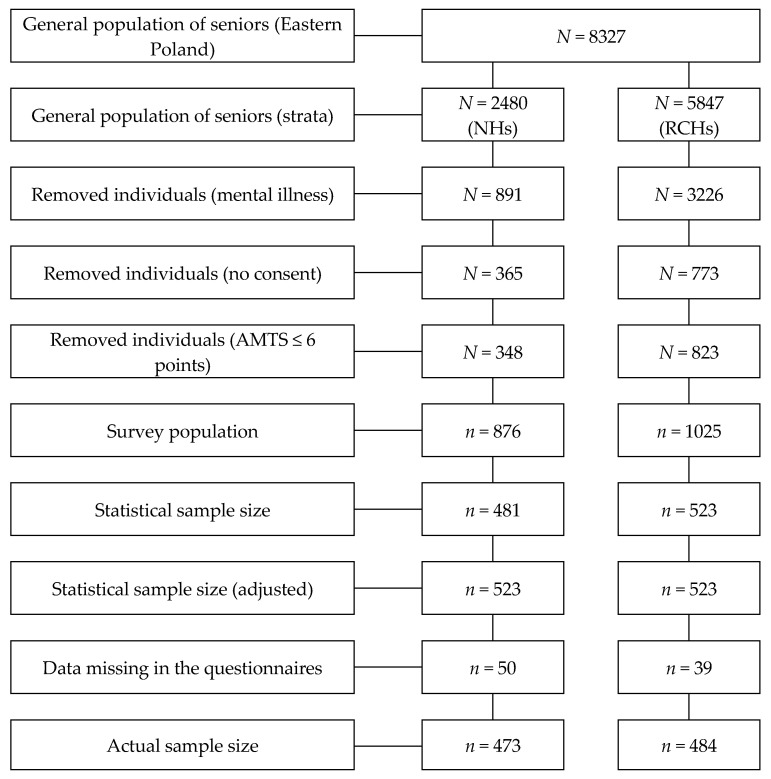
Stages of selection of respondents to participate in the study.

However, for the purpose of this study, equal sample sizes (*n* = 523) were adopted in order to ensure comparability of results for the assumed maximum error rate. The sample size of NHs respondents was adjusted to the size of the RCHs sample (Figure 1). The collected questionnaires were verified—89 of them contained missing data (unanswered questions).

The study protocol was approved by the Bioethics Committee of the Medical University of Lublin (KE−0254/86/2015) and informed written consent was obtained from all participants.

Selection criteria:

1. age of ≥ 60 years;
2. having a mental state which allowed for the filling in of the questionnaires;
3. having written confirmation of willingness to participate in the study.

Basic descriptive statistics are presented in Table 1.

### 2.2. Method and Questionnaires

The study was based on a quantitative strategy—a cross-sectional survey method. The measurement of variables was carried out on the basis of standardised tools:

a. WHOQoL-BREF questionnaire—is a shorter variant of the WHOQoL-100 scale which allows to determine the subjective quality of life. The scale was created on the basis of the HRQoL concept. It contains 26 questions which relate to the following components of the quality of life:
  ▪ general quality of life (question number 1)
  ▪ general health status (question number 2)
  ▪ physical domain (questions 3, 4, 10, 15, 16–18)
  ▪ psychological domain (questions 5–7, 11, 19, 26)
  ▪ social domain (questions 20–22)
  ▪ environmental domain (questions 8, 9, 12–14, 23–25)
  The questions of questionnaires 1 and 2 were subjected to a separate analysis. The scores for individual domains range from 4 to 20, and from 1 to 5 for questions number 1 and 2. The interpretation of results has a positive direction, whereby the higher the score obtained by the respondent, the better his/her quality of life [23].
b. Social Support Scale—the questionnaire was used to measure social support in functional terms (functional support). The scale is based on the concept of social support assuming the existence of its specific types. It consists of 24 questions and provides information about general support and four types of support:
  ▪ informational support (questions 1–6);
  ▪ instrumental support (questions 7–12);
  ▪ appraisal support (questions 13–18);
  ▪ emotional support (questions 19–24).
  The overall score falls within the range of 24 and 120 points and each type of support falls within the range of 6 and 30 points. The interpretation of results has a negative direction—the higher the score, the lower the functional support and its individual types [24].
c. The Courage Social Network Index—the questionnaire was used to measure social support in structural terms (structural support). The questionnaire was created on the basis of a theoretical model of the function of informal networks. It consists of five questions related to different sources of support: Partner, parents, children, grandchildren, relatives, co-workers, neighbours and friends. The overall score ranges from 0 to 100%. Higher percentages correspond to higher structural support (high number of strong emotional ties as well as frequent direct relations with members of the support network). The interpretation of results has a positive direction [25,26].
d. The Hodgkinson’s Abbreviated Mental Test Score was used to assess the cognitive functions of the seniors. The questionnaire consists of 10 questions and instructions addressed to the respondents. Respondents received 1 point for each time they gave a correct answer or followed an instruction. The score of 6 or less points disqualified the respondent from the study.

### 2.3. Data Analysis

Statistical analyses were carried out through IBM SPSS Statistics v23. The classic threshold of α = 0.05 was assumed as the statistical significance level. Basic descriptive statistics and tests of the compliance of distributions with the Gauss curve took place after the elimination of outliers. In the case of dependent variables, the skewness value did not exceed the absolute value of 1.0. Therefore, statistical analyses were performed with the use of parametric tests.

## 3. Results

### 3.1. Verification of Hypothesis 1

The first step was to verify the existence of a linear correlation between social support and the perception of general quality of life, and general health status. The results showed that all correlations in the group of respondents staying in NHs were statistically significant (*p* < 0.001) and had a negative direction. The above does not apply to structural support, which had positive correlations. Better assessment of social support was accompanied by better general quality of life and health status. However, their presence was mostly small (*r* < 0.3). For the older people staying in RCHs, the general quality of life and health status correlated negatively and poorly with functional support (*r* < 0.3). The higher the assessment of social support, the better the assessment of general quality of life and health status. The correlations with a positive direction was the one between structural (NHs and RCHs) and emotional support (RCHs) and general quality of life and health status. However, most these correlations were not statistically significant (Table 2).

The next step was to verify the interdependence between social support and individual domains of the quality of life. In the case of respondents from NHs, structural support showed a positive correlation. However, all correlation coefficients of functional support had a negative direction and were statistically significant (*p* < 0.001). With the increase of social support, a simultaneous increase in the scores for particular domains of the quality of life was observed (Table 3). Most correlations were moderately strong (*r* > 0.3). The same analyses performed in the group of respondents from RCHs revealed fewer statistically significant correlations. However, structural support correlated with every domain of the quality of life. On the other hand, functional, informational and instrumental types of support correlated with psychological, social and environmental domains (*p* < 0.001). The assessment of functional support and its types correlated negatively with the quality of life—the better the assessment of support, the higher the assessment of quality of life. This statement did not apply to emotional support. In the case of respondents from RCHs, this type of support correlated positively with physical, psychological and social domains. However, the relationships for the last two domains were not statistically significant (*p* > 0.05). This means that a better perception of the physical domain was associated with a decrease in the need for emotional support (Table 3).

Hypothesis 1 was partially confirmed by the statistical analyses. It was shown that there was a linear correlation between social support and the perception of the quality of life in most of the analysed categories of variables. The correlation between the variables was most visible among the respondents from NHs.

### 3.2. Verification of Hypothesis 2

Using the Student’s test, it was verified whether the type of care institution altered the quality of life (Table 4). Average values in the group of respondents from RCHs were higher in each of the quality of life domains in comparison with respondents from NHs. A strong effect of differences was observed for the physical domain. On the other hand, the effect was average for the social domain. Other differences were rather small (*d* < 0.5).

A significant differentiation of social support depending on the type of institution concerned all categories of support (*p* < 0.001). Comparable average values showed that respondents from RCHs gave higher scores to structural and functional support and all of its types. The strength of the effect of differences was weak in the case of informational support (*d* < 0.5) and moderately strong in the case of other categories of support (*d* < 0.5). Structural support achieved the strongest effect. The results are presented in Table 5.

As a result of statistical analyses, Hypothesis 2 was fully confirmed. It was found that the type of care institution differentiated both the level of social support and self-assessment of the quality of life of seniors. The perception of these variables was higher in the opinions of respondents from RCHs.

## 4. Discussion

### 4.1. Functional Support and Quality of Life

In our study, social support correlated significantly with the seniors’ quality of life. Similar results were obtained by Kurowska and Kajut [19] and Vitorino et al. [20]. In this study, the respondents from RCHs who maintained strong family ties and social relations with other residents of the institution gave higher scores to their quality of life. This is understandable given the sustained effect of support, which enabled seniors to meet their needs for closeness and safety. Drageset et al. [2] conducted a cross-sectional study on respondents from NHs. They proved that social relations of an emotional nature with significant others were an important element of the psychological sphere of the seniors’ quality of life. Our study seems to confirm this correlation, as respondents with more extensive support networks and those who experienced different types of support also had a better self-assessment of the psychological state. de Belvis et al. and Garcia et al. [8,9] observed a strong correlation between the physical and mental states and the level of social support. The respondents, who were more often visited by family members and friends, were characterised by having better functioning in these domains. Moreover, living at a significant distance from the closest relatives lowered the health potential of seniors. It could be related to the insufficient level and type of support, and thus lead to a decrease in the self-care motivation. This finding is confirmed by the results of our study. The physical and psychological domains of the quality of life depend on the level of support received. This trend was very clear in the case of respondents from NHs. The more stable and extensive the support network, the greater the awareness and possibility of real help [12]. Chang et al. [13] report that support from family and other close people is positively correlated with domains of the quality of life. The results of their study show that seniors received limited social support from their relatives and friends. Therefore, they assessed the support as insufficient. The authors explain this conclusion by the marital status of respondents—most of them were not in a formal relationship or were divorced/widowed. Moreover, seniors with a low socio-economic status who live alone, often decide to be placed in a care institution.

### 4.2. Structural Support and Quality of Life

The studies conducted by Street et al. [10] as well as Kurowska and Kajut [19] show an interesting correlation among life satisfaction and social relations among people in a care institution. The results suggest that seniors who do not have, or do not maintain, any family ties, showed a stronger ability to create new relations than to continue the existing ones. It is possible that many seniors had lived alone before moving to a care facility. Institutional care and the associated stay in a larger group of people were perceived by respondents as a positive phenomenon. The most important aspect for the respondents self-assessment of the quality of life was support from their neighbours, followed by support by their family and life partners. On the other hand, contacts with the family did not have a significant impact on the quality of life. Respondents thought that positive relations within the institution (between the staff and other seniors) were the most valuable. These results once again confirm the assumption that seniors, despite experiencing a stressful situation when changing the environment, are able to create new relations in the institution. They valued these new relations more than the existing ones. In our opinion, the lack of contacts with relatives and being placed in a care facility are not always associated with a limited social integration. This is confirmed by the results of a study conducted by Andrew et al. [5]. Satisfactory social contacts from the previous years are associated with a positive self-assessment of the quality of life. Emotional support received from staff has a significant impact on the life experiences of respondents living in long-term care institutions. In order to shape social networks, long-term care institutions can provide opportunities for socialisation and encourage interaction between residents and the staff. They can also provide residents with more opportunities to interact with family and friends [4,7,15]. According to Ye and Zhang [12], together with economic development and the accompanying changes in the social structure, seniors in rural areas are nowadays less dependent on the natural environment, and they can still maintain relations between members of their social networks. Seniors are more likely to interact with friends and develop on a social level. The authors pointed out that older people with limited family relations had a worse self-assessment of their physical health than those with non-family relations. The reason for this correlation may be that friends can provide more relevant types of support (informational and emotional) that are beneficial to mental health. Members of the family network, on the other hand, tend to provide more tangible support beneficial to physical health. The results of other studies confirm a correlation between the quality of life and the type of the received social support [10,11,12,13,20,27]. Social support and quality of life were the subject of one of the studies conducted by Kurowska and Błaszczuk [14]. According to the authors, higher scores for social support were accompanied by better quality of life of the respondents. The above is fully supported by the results of this study, in which the components of the quality of life correlated with the functional dimension of support and its different types. Vogel et al. [28] also noticed similar correlations, but in relation to respondents who were not covered by round-the-clock care in an institution. The results of these studies confirmed that older people experiencing functional support adequate to their needs are more resistant to stress, which in turn allows them to cope with a range of problems. On the other hand, the results presented by Çimen and Akbolat [6] show a strong impact of the perceived social support, especially from family members, on life satisfaction. Our study revealed that a better perception of the physical domain was associated with a decrease in the need for emotional support. This was confirmed by other studies [6,9,12,20]. The decrease in the need for emotional support can be explained by the gradual adaptation of the senior to institutional conditions or by the creation of psychological adaptive mechanisms [29].

### 4.3. Nursing Homes vs. Residential Care Homes

Respondents from RCHs were more satisfied with their quality of life than those living in NHs. Apart from that, satisfaction with the received support was also higher among seniors living in RCHs. On the one hand, the difference in results could have been caused by a different duration of stay in NH than in RCH. Due to the fact that the respondents usually stayed in NHs for a certain period of time, they returned to the natural environment after the end of the post-hospitalisation treatment. On the other hand, respondents requiring care of a nursing nature (NH) were in worse shape than the respondents staying in RCHs. In addition, RCH, as an institution of a social nature, serves a number of functions aimed at sustaining the socialisation process. It provides its residents with the opportunity to establish new, or maintain, existing social relationships. It also plays an integrating role, allowing for the possibility of integrating with other residents and the local community [4,5,7,30]. Therefore, respondents staying in RCHs achieved statistically better scores in most domains of the quality of life than respondents staying in NHs. Other consistent results are presented by Chou et al. [31]. Respondents from RCH institutions were more satisfied with social relations than seniors in NH institutions. Top and Dikmetaş [30] and Andresson et al. [32] explain that respondents staying in RCH institutions were more independent and thus more mobile than seniors receiving nursing care (NH). The latter, due to their health status, were highly dependent on the help of medical staff and they spent most of their time in bed. Therefore, the perception of their quality of life was at a lower level.

### 4.4. Study Limitations

There are some limitations worth mentioning in this study. It seems reasonable to use a tool to measure variables in the senior’s population, such as an age-specific quality of life questionnaire. Furthermore, due to the change of residence, it seems important to measure the objective dimension of the quality of life (e.g., living conditions, financial resources, extent to which needs are met or duration of stay in the care of a given facility). An important element of social support for older people in the care of an institution are care workers. In the case of some respondents, the group of professional carers becomes a part of their personal support network. Therefore, this group should be taken into account in further studies on social support.

### 4.5. Recommendations for Long-Term Nursing Practice

In order to develop the long-term nursing practice, it seems reasonable to conduct a study based on a qualitative strategy, which could complement results collected in quantitative research. This would allow deeper and comprehensive analysis of the quality of life of older people in the context of social relationships. Research on the quality of life and social support allows to see the problems experienced by an elderly person, and thus to take actions aimed at solving them, e.g., increasing the level of quality of care provided. Any changes that stimulate the increase in demand for institutional care of a stationary nature can be an incentive for organizers of health care and social assistance to specify, and consolidate, the role of geriatrics in contemporary society, and determine the working methods that are adequate to the needs of comprehensive long-term care and institutional assistance.

## 5. Conclusions

Social support is a significant component in the perception of the quality of life for seniors. Its variable level determines the subjective assessment of functioning in particular domains of the quality of life. RCH as an institution of a social nature satisfies the needs for support at a higher level than NH, which translates into a better perception of the senior’s quality of life. Emotional support proved most valuable for most seniors, regardless of the type of care institution. In the long-term, it seems justified to create conditions similar to those of the natural environment and to involve the NH/RCH staff in care for the seniors. These activities are necessary to maintain an optimal level of social functioning among the seniors in an institutional environment.

## Figures and Tables

**Table 1:** Basic descriptive statistics.

Variables	NH				RCH			
	M	SD	sk.	kurt.	M	SD	sk.	kurt.
General quality of life	3.34	0.84	−0.22	−0.09	3.42	0.90	−0.60	0.66
General health status	2.93	0.98	−0.05	−0.80	3.24	1.00	−0.08	−0.51
Physical	12.00	2.84	0.05	0.07	14.44	2.89	0.00	−0.79
Psychological	12.13	2.74	0.05	−0.54	13.33	2.77	0.43	−0.34
Social	12.23	2.49	−0.24	−0.26	13.71	2.56	0.19	−0.19
Environmental	13.83	2.47	−0.17	−0.22	14.08	2.33	0.06	−0.09
Structural support	40.42	24.65	−0.24	−0.95	63.38	11.02	−0.29	−0.17
Functional support	47.07	12.51	0.76	0.19	39.83	10.62	1.13	1.31
Informational support	11.00	3.34	0.59	0.06	9.83	3.30	0.84	0.24
Instrumental support	13.22	3.92	0.42	−0.25	11.19	3.44	0.59	0.15
Appraisal support	13.62	3.78	0.44	−0.05	11.12	3.55	1.01	1.03
Emotional support	9.23	3.30	1.10	0.71	7.69	2.60	2.10	4.36

M—mean, SD—standard deviation, sk.—skewness, kurt.—kurtosis.

**Table 2:** Correlation between social support and general quality of life and health status.

Type of Social Support	Test	General Quality of Life		General Health Status	
		NH	RCH	NH	RCH
Structural support	*r*	0.21	0.22	0.08	0.19
	*p*	<0.001	<0.001	0.084	<0.001
Functional support	*r*	−0.33	−0.19	−0.21	−0.16
	*p*	<0.001	<0.001	<0.001	0.001
Informational support	*r*	−0.20	−0.08	−0.12	−0.12
	*p*	<0.001	0.070	0.009	0.010
Instrumental support	*r*	−0.24	−0.34	−0.11	−0.17
	*p*	<0.001	<0.001	0.016	<0.001
Appraisal support	*r*	−0.31	−0.01	−0.19	−0.08
	*p*	<0.001	0.807	<0.001	0.097
Emotional support	*r*	−0.27	0.01	−0.16	0.00
	*p*	<0.001	0.755	0.001	0.928

*r*—Pearson correlation coefficient, *p*—statistical significance.

**Table 3:** Correlation between social support and domains of the quality of life.

Type of Social Support	Test	Physical		Psychological		Social		Environmental	
		NH	RCH	NH	RCH	NH	RCH	NH	RCH
Structural support	*r*	0.21	0.10	0.33	0.20	0.30	0.30	0.24	0.36
	*p*	<0.001	0.035	<0.001	<0.001	<0.001	<0.001	<0.001	<0.001
Functional support	*r*	−0.39	−0.03	−0.46	−0.17	−0.38	−0.15	−0.48	−0.24
	*p*	<0.001	0.492	<0.001	<0.001	<0.001	0.001	<0.001	<0.001
Informational support	*r*	−0.28	−0.07	−0.31	−0.20	−0.25	−0.15	−0.32	−0.24
	*p*	<0.001	0.107	<0.001	<0.001	<0.001	0.001	<0.001	<0.001
Instrumental support	*r*	−0.32	−0.11	−0.34	−0.28	−0.34	−0.19	−0.37	−0.33
	*p*	<0.001	0.020	<0.001	<0.001	<0.001	<0.001	<0.001	<0.001
Appraisal support	*r*	−0.34	−0.03	−0.44	−0.04	−0.25	−0.05	−0.42	−0.11
	*p*	<0.001	0.481	<0.001	0.337	<0.001	0.253	<0.001	0.013
Emotional support	*r*	−0.24	0.15	−0.38	0.06	−0.29	0.01	−0.38	−0.03
	*p*	<0.001	0.001	<0.001	0.211	<0.001	0.905	<0.001	0.525

*r*—Pearson correlation coefficient, *p*—statistical significance.

**Table 4:** Comparison of the quality of life depending on the type of care institution.

Quality of Life	NH		RCH		Test		95% CI		d
	M	SD	M	SD	t	*p*	LL	UL	
General quality of life	3.34	0.84	3.42	0.90	−1.55	0.121	−0.20	0.02	0.10
General health status	2.93	0.98	3.24	1.00	−4.85	<0.001	−0.43	−0.18	0.31
Physical	12.00	2.84	14.44	2.89	−13.18	<0.001	−2.80	−2.08	0.85
Psychological	12.13	2.74	13.33	2.77	−6.78	<0.001	−1.56	−0.86	0.44
Social	12.23	2.49	13.71	2.56	−9.02	<0.001	−1.79	−1.15	0.58
Environmental *	13.83	2.47	14.08	2.33	−1.57	0.117	−0.55	0.06	0.10

M—mean, SD—standard deviation, t—Student’s t-test, *p*—statistical significance, LL—lower limit, UL—upper limit, *d*—Cohen’s effect size, *—unequal variances (Welch correction was applied).

**Table 5:** Comparison of social support depending on the type of care institution.

Social Support	NH		RCH		Test		95% CI		*d*
	M	SD	M	SD	t	*p*	LL	UL	
Structural support *	40.42	24.65	63.38	11.02	−18.53	<0.001	−25.40	−20.53	1.21
Functional support *	47.07	12.51	39.83	10.62	9.58	<0.001	5.76	8.73	0.63
Informational support	11.00	3.34	9.83	3.30	5.39	<0.001	0.74	1.59	0.35
Instrumental support *	13.22	3.92	11.19	3.44	8.46	<0.001	1.56	2.51	0.55
Appraisal support	13.62	3.78	11.12	3.55	10.36	<0.001	2.01	2.95	0.68
Emotional support *	9.23	3.30	7.69	2.60	7.95	<0.001	1.17	1.93	0.52

M—mean, SD—standard deviation, t—Student’s t-test, p—statistical significance, LL—lower limit, UL—upper limit, d—Cohen’s effect size, *—unequal variances (Welch correction was applied).

## Abbreviations

NH	nursing homes
RCH	residential care homes
HRQoL	Health Related Quality of Life
WHOQoL-BREF questionnaire	The World Health Organization Quality of Life BREF questionnaire
